# Depression and homonegativity disparities in Brazilian gay men: The importance of HIV-status

**DOI:** 10.1177/13591053251383743

**Published:** 2025-10-29

**Authors:** Iara Teixeira, Felipe Alckmin-Carvalho, Guilherme Welter Wendt, António Oliveira, Lucia Nichiata, Henrique Pereira

**Affiliations:** 1 Psychology Research Center, School of Psychology, University of Minho, Braga, Portugal; 2 Department of Psychology and Education, Faculty of Social and Human Sciences, University of Beira Interior, Covilhã, Portugal; 3 School of Nursing, University of São Paulo, Brazil; 4 Health Sciences Center, Department of Medicine and Postgraduate Program in Applied Health Sciences, State University of Western Paraná (UNIOESTE), Francisco Beltrão (PR), Brazil; 5 Research Center in Sports Sciences, Health Sciences and Human Development (CIDESD), Vila Real, Portugal

**Keywords:** HIV, depression, homonegativity, stigma, mental health

## Abstract

This study examines the association between HIV status, depressive symptoms, and homonegativity among Brazilian gay men. A total of 410 participants, 217 (52.9%) living with HIV and 193 (47.1%) without HIV, completed the Beck Depression Inventory II, the Internalized Homophobia Scale, and a sociodemographic questionnaire. Results indicated high prevalence of depressive symptoms in both groups (52.2% among those living with HIV and 62.6% among those without). Mean BDI-II scores were lower among participants living with HIV (*M* = 11.48, SD = 9.00) compared to those without HIV (*M* = 13.92, SD = 9.62; *p* = 0.008). Homonegativity also differed, with lower scores among participants living with HIV (*M* = 25.78, SD = 8.83) than those without HIV (*M* = 30.92, SD = 3.65; *p* < 0.001). Homonegativity contributed to the association between HIV status and depression. These findings highlight the need for policies against stigma, expanded mental health care, and tailored interventions.

## Introduction

The HIV epidemic in Brazil is said to be concentrated, with its burden affecting specific groups of populations such as gay and other men who have sex with men (MSM), transgender people, and commercial sex workers (World Health Organization, 2024a). MSM globally are 26 times more likely to acquire HIV compared to the general population (WHO, 2024b). Different studies have shown that the stigma of homosexuality has been legitimized by religious and cultural narratives (Alckmin-Carvalho et al., 2024a; Wasmuth et al., 2021). For instance, Brazil, a country with nearly 90% Christians (Instituto Brasileiro de Geografia e Estatística, 2010), is one of the Western countries with the highest rates of violence against persons in the LGBTQIA+ community (Associação Nacional de Travestis e Transexuais, 2025; Cerqueira and Bueno, 2023).

There is ample evidence of the psychological consequences of homonegativity and internalized stigma (Jodelet, 1989; Nguyen et al., 2024; Sulistina et al., 2024). Internalized homonegativity refers to the process by which individuals adopt and direct toward themselves the negative societal attitudes about homosexuality (Meyer, 2003; Szymanski et al., 2008). In contrast, perceived community stigma concerns the perception that one’s social environment holds hostile or discriminatory beliefs toward gay men as a group (Earnshaw and Chaudoir, 2009; Herek, 2009). These related but distinct constructs capture different levels of stigma, one internal and one external, that together shape psychological outcomes. Furthermore, many gay men living with HIV are exposed to layered stigma, in which sexual orientation stigma intersects with HIV-related stigma and, in some cases, additional vulnerabilities such as poverty or racial discrimination, creating compounding risks to mental health (Earnshaw et al., 2013; Logie and Earnshaw, 2015).

People reporting high internalized sexual stigma often feel shame and guilt, which has been associated with low self-esteem, mental health complaints, loneliness, restricted social networks, and fear of intimacy, resulting in less fulfilling relationships (Guzmán-González et al., 2023; O’Donnell and Foran, 2024). Both internalized and community-derived homonegativity are associated with psychological distress, including mood and anxiety disorders and substance use problems (Lattanner et al., 2025; Newcomb and Mustanski, 2010). Internalized homonegativity and internalized stigma occur when someone adopts the negative attitudes and prejudices of society toward their own sexual orientation or HIV status, which, in turn, are defined as community-derived forms of internalized homonegativity and general stigma associated with HIV.

Internalized homonegativity is associated with rejection sensitivity, whereby neutral social experiences are perceived as hostile, which relates to withdrawal and emotional disturbance, suggesting preclinical depression (Petruzzella et al., 2020). Evidence suggests gay men living with HIV experience higher depression levels than those not living with HIV. In Brazil, HIV-related stigma has been consistently linked to delays in treatment initiation and to high prevalence of depression and anxiety among people living with HIV, particularly when mental health support is limited (Nomoto et al., 2015; Reis et al., 2017). Structural barriers such as socioeconomic inequalities and regional disparities in health services also contribute to restricted access to antiretroviral therapy (Cunha et al., 2025)

HIV is now considered a chronic condition, and its management has become largely manageable, particularly when individuals are diagnosed and treated early (Calazans et al., 2023; Raubinger et al., 2022). The pharmacological development of antiretroviral drugs has led to reduced toxicity and simpler treatment regimens, establishing that achieving an undetectable viral load eliminates the risk of HIV transmission (Benzaken et al., 2019). Consequently, the quality of life for people living with HIV has significantly improved in recent decades (Alckmin-Carvalho et al., 2024b). Moreover, pre-exposure prophylaxis (PrEP) has emerged as a powerful HIV prevention strategy, particularly for gay men and transgender women in Brazil, significantly reducing new infections and empowering them with autonomy over their sexual health (Pena et al., 2023). Nonetheless, there are very clear differences in the quality of care offered to those living with HIV, reflecting internal regional disparities within the country.

HIV-related stigma is still widespread in Brazil (Alckmin-Carvalho et al., 2024c; Figueiredo Catelan et al., 2022; Monteiro et al., 2012). Gay men living with HIV usually experience what is known as “double stigma,” that is, being discriminated against due to both sexual orientation and serophobia (i.e. fear of HIV; Alckmin-Carvalho et al., 2023; Allan-Blitz et al., 2021). The double burden of sexual orientation stigma and stigma associated with HIV has been linked to greater pre-existing vulnerabilities (Meyer, 2003), such as social isolation, especially in more conservative areas, which can further affect mental health (Berg and Ross, 2014; Cohen, 1988).

Despite recent legal advances such as same-sex marriage and the ban on conversion therapy in Brazil (Aragusuku and Lara, 2019; Figueirêdo, 2021), homonegativity remains pervasive. For example, Alckmin-Carvalho et al. (2023) assessed perceived and internalized homonegativity in gay men in Brazil and concluded that 93% of the gay men believed that Brazilian society punishes gay men and 98.55% believed that discrimination against members of the LGBTQIA+ community still exists in Brazil. The recent resurgence of far-right religious parties with significant political power strongly endangers the recently achieved rights of the LGBTQIA+ community. Moreover, extremist religious groups employed in politics sanction LGBTQIA+ discrimination and violence under the guise of freedom of expression both in Brazil (Boppana and Gross, 2019) and in other nations, such as the United States (Stern, 2024) and Portugal (Fernandes et al., 2023).

The blending of political and religious aspects contributes to enduring power and oppression dynamics against sexual minorities in Brazil (Mezacasa and Lima Junior, 2022). For example, since childhood, Brazilian gay men tend to be socialized and subjected to content that renders homosexuality lesser, immoral, and even pathological (Alckmin-Carvalho et al., 2024a). A strong influence of religious beliefs, combined with high rates of violence against the LGBTQIA+ population also occur while Brazil has a health system that is globally recognized for serving people living with HIV (Azevedo et al., 2024).

Studying factors influencing the mental health of gay men with and without HIV can clarify their challenges and inform health service practices. Consequently, the present study aims to investigate the prevalence and factors associated with depression among gay men living with and without HIV in Brazil. Specifically, the investigation sought to compare the severity of depressive symptoms between these two groups, while also analyzing potential differences among the items of a widely used instrument (i.e. Beck Depression Inventory [BDI-II]; Beck et al., 1996). In addition, the relationships between different types of stigma, depressive symptoms, and homonegativity were explored. Based on previous studies, it was hypothesized that homonegativity would be associated with depressive symptoms (Liu and Ren, 2024) and with both types of stigma (Wasmuth et al., 2021). Although no assumption was made regarding sorological status with depression, a possible indirect effect via homonegativity was explored (Liu and Ren, 2024; Wasmuth et al., 2021).

## Method

This is an observational, cross-sectional study. The investigation was conducted at the School of Nursing,University of São Paulo.

### Participants and procedures

The study included Brazilian gay men living with and without HIV. Participants had to self-identify as cisgender gay men, aged 18 or older, with internet access and with the possibility to respond to the survey with privacy. Bisexual and transgender men were not included in our study because we believe that the homonegativity and depression they face have particularities that require exclusive investigation for each gender and sexual orientation subgroup. Homen Brazilian gay men living abroad were also excluded from the study.

Due to the high levels of HIV-related stigma and mental health stigma in Brazil, individuals living with HIV are generally reluctant to disclose their serostatus or psychological difficulties. Because our study was not conducted within a healthcare setting and we did not have access to registries of potential participants, conventional recruitment strategies would have been insufficient to reach this population. For this reason, we employed a snowball sampling strategy, starting with a small group of seed participants and expanding through their social and community networks, where mutual trust facilitated participation. This approach reduced barriers related to invisibility and fear of discrimination, and allowed us to access individuals who might otherwise not have participated. We deliberately avoided relying exclusively on participants who had publicly disclosed their HIV status on online support networks (e.g. Instagram or Facebook), since such an approach could generate selection bias and fail to represent the broader population of gay men living with HIV in Brazil.

The selection of gay men living with HIV took place through an approach to two nurses who were Ph.D. students of the School of Nursing at the University of São Paulo, São Paulo, Brazil. The professionals assisted in identifying the first five candidates (seeds). Five more seed participants were found through social media like Instagram and Facebook, on support pages or internet communities aimed at gay men living with HIV. The seed participants, who were selected because they had ample access to gay men living with HIV in their personal contacts and social networks, completed the psychological assessment instruments and were instructed to share the research link with their social networks and to make direct contact with close people living with HIV. They were also instructed to ask these new participants to share the research with their contacts. Participants were told that the survey was anonymous and that there was no risk or reward for participating. No financial compensation was provided for participation. The instruments took an average of 15 minutes to complete.

For the group of men not living with HIV, recruitment relied on a combination of strategies. Participants with unknown HIV serology were recruited directly from the researchers’ institutional and personal social networks, and the survey asked: “Have you tested positive for HIV?.” Those who answered negatively or did not report a positive test were included in this group. In addition, some participants were reached through public posts on social media covering a broad range of topics, unrelated to HIV. This mixed strategy allowed us to diversify recruitment and to avoid concentrating the sample only among individuals connected to HIV-related networks. Of those recruited via the researchers’ institutional and personal networks, 15% reported living with HIV and were reclassified into the group living with HIV for analysis.

**Figure 1.**
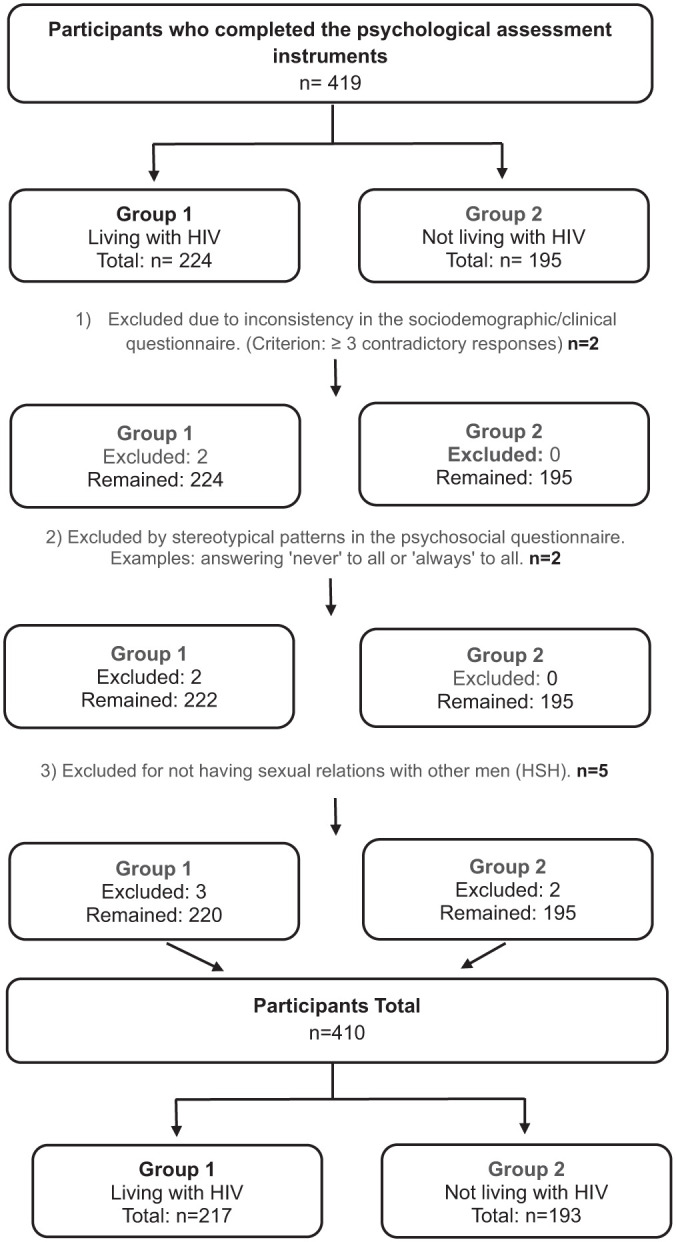
Flowchart for selection and composition of participant groups.

To ensure data integrity, quality control procedures were applied. Participants with inconsistent responses, stereotypical answering patterns, or who did not report sexual relations with men were excluded. In total, five participants were removed. The full process of sample composition and exclusions is detailed in Figure 1.

The sample size was estimated using IBGE data on the male population and national HIV prevalence among gay men. According to the IBGE (2022), in the last census, Brazil had approximately 1.8 million (1.82%) self-declared gay men. Considering that the prevalence of HIV among gay men is around 18.4% (Kerr et al., 2018), a minimum of 174 participants living with HIV was targeted to achieve a 90% confidence level and 5% margin of error. Using OpenEpi (Sullivan et al., 2019), this target corresponded to a statistical power of 99%. Additionally, a post-hoc power analysis conducted with G*Power (Faul et al., 2007), based on the observed average effect size among continuous variables (depression, homonegativity, and stigma; *d* = 0.67) and the ratio between groups, confirmed that the achieved power was 99.99%.

### Measurement Instruments

*Internalized Homophobia Scale* (Ross and Rosser, 1996): We used the 19-item Brazilian version of the scale, adapted and validated by Lira and Morais (2019). In the Brazilian version, some items from the original version were removed due to low psychometric performance, resulting in a shorter scale with two factors. It comprises two factors: internalized homonegativity and the perception of homonegativity in the community. It uses a 5-point Likert scale (1 - strongly disagree to 5 - strongly agree). Examples of the statements in the scale are: (1) Typically, effeminate gay men make me feel uncomfortable; (2) I prefer to have anonymous sexual partners; and (3) Life would be easier if I were heterosexual. Although the scale doesn’t have a cut-off for classifying levels of homonegativity, higher scores indicate higher levels of internalized homophobia (Lira and Morais, 2019). Cronbach’s alpha was 0.814 for the internal perception of stigma and 0.622 for the external perception of stigma.

*Beck Depression Inventory* (BDI-II; Beck et al., 1996): It is composed of 21 items that cover cognitive, affective, and physical manifestations of depressive symptoms. Each item consists of four statements indicating symptom severity, scored from 0 (I don’t feel sad) to 3 (I’m so sad or unhappy that I can’t stand it). Levels of depression are categorized based on the total score: 0–11 = minimal, 12–19 = mild, 20–35 = moderate and 36–63 = severe (Beck et al., 1996). We used the validated Brazilian version of the BDI-II, which demonstrated good psychometric properties (α = 0.81) in its validation study (Gomes-Oliveira et al., 2012). In the current study, an even higher coefficient was found (α = 0.90).

*Sociodemographic Questionnaire*: This instrument, developed by the authors, collects information on participants’ sociodemographic characteristics, including age, employment status, education level, HIV status, housing conditions, living arrangements and income. Although route of HIV acquisition was not assessed, national estimates indicate that vertical transmission in Brazil is very low (below 2%; Ministério da Saúde, 2025), making it highly likely that the majority of cases in this sample acquired HIV through horizontal transmission.

### Ethical Aspects

This study was approved by the Research Ethics Committee of the School of Nursing,University of São Paulo (number: 4.601.952, CAAE: 31527820.7.0000.5392; 19 March 2021). All the participants provided written informed consent. The informed consent form was presented on the first page of the online research protocol. Only participants who provided electronic consent were able to proceed to the questionnaires.

### Data Analyses

Statistical analyses were carried out using SPSS software (v. 29). Initially, descriptive statistics were conducted both to characterize the sample and to analyze the participants’ depression and homonegativity scores, including means, standard deviations, frequencies and percentages. For univariate analyses, the normality of the data was assessed via the inspection of skewness and kurtosis (Burdenski, 2000). In other cases, normality was checked by using the Shapiro-Wilk test. Moreover, the homogeneity of the variances was checked using the Levene’s test.

The chi-square test, along with the calculation of effect sizes (Cramer’s V), was used to analyze the differences in sociodemographic data and the severity of depressive symptoms between the groups of participants living with and without HIV. Student’s t-test, along with Cohen’s d as a measure of effect size, were used to verify the differences between the groups for each symptom of depression and also for the BDI-II total scores, homonegativity, internal perception of stigma and external perception of stigma. Pearson’s correlations were computed to explore the factors associated with depressive symptoms in the sample.

Effect sizes considered the criteria proposed by Cohen (1988). For Cohen’s d, the following values were considered: small (0.2–0.5), medium (0.5–0.8) and large (>0.8); and for Cramer’s V, small (<0.30), medium (<0.50) and large (>0.50). In addition, analyses of indirect effects were carried out using the PROCESS macro for SPSS (Hayes, 2018), with the aim to explore whether homonegativity may account for the relationship between HIV status and depression.

## Results

A total of 410 gay men participated in the study. Most had higher education or above (80.7%), were employed (83%), and lived with family members or partners (55%). Regarding housing, 44.1% owned their home and 49.0% rented, with no significant differences by HIV status. About one-third (33.9%) lived alone. Most participants earned between 1 and 7 minimum wages (44.6% in the 1–3 range; 30.8% in the 4–7 range), while 20% reported incomes above 8 minimum wages.

Chi-square tests (Table 1) revealed significant differences by HIV status in education, employment, and income. Participants not living with HIV more often had higher education and were employed, whereas those living with HIV were more likely to have completed only high school, be unemployed, or on sick leave (χ² = 8.580, *p* = 0.03, *V* = 0.14). Income disparities were more marked (χ² = 29.365, *p* = 0.001, *V* = 0.28), with participants living with HIV concentrated in lower income brackets and those not living with HIV more represented in higher income categories.

**Table 1.** Sociodemographic characteristics by HIV status..

	Total (*n* = 410)		Living with HIV (*n* = 217)		Not living with HIV (*n* = 193)		Chi-square	*p*-value	Cramer’s V
Variables	*n*	%	*n*	%	*n*	%			
Educational level									
Up to high school or technical training	79	19.3	64	29.4	15	7.7	30.982	0.001	0.27
Higher education and above^a^	331	80.7	153	70.5	178	92.2			
Employment status									
Employed	340	82.9	171	78.8	169	87.6	8.580	0.03	0.14
Unemployed	51	12.4	31	14.3	20	10.4			
On sick leave	14	3.4	10	4.6	4	2.1			
Retired	5	1.2	5	2.3	-	-			
Housing condition									
Own	181	44.1	95	43.8	86	44.6	1.578	0.45	-
Rented	201	49.0	104	47.9	97	50.3			
Borrowed/provided	28	6.8	18	8.3	10	5.2			
Living arrangements									
With family/partner	228	55.6	117	53.9	111	57.5	0.858	0.65	-
With colleagues/friends	43	10.5	22	10.1	21	10.9			
Alone	139	33.9	78	35.9	61	31.6			
Monthly income (in multiples of the Brazilian minimum wage)^b^									
1–3	168	44.6	106	52.0	62	35.8	29.365	0.001	0.28
4–7	116	30.8	62	30.4	54	31.2			
8–11	42	11.1	25	12.3	17	9.8			
12–15	22	5.8	7	3.4	15	8.7			
>15	29	7.7		2.0	25	13.0			

a In the Brazilian education system, “higher education” refers to any post-secondary degree program, including undergraduate (bachelor’s), graduate (master’s and doctoral) studies.

b In 2024, the minimum wage in Brazil was BRL 1412 (EUR 229.25; USD 249.02). Values converted using the exchange rate of 26 Dez 2024.

The comparative analysis did not show statistically significant differences between the levels of depression (minimal, mild, moderate, severe) in participants living with HIV and without HIV (χ^2^ = 6.119, *p* = 0.10). Therefore, no statistically significant relationships were identified. The analysis of the continuous BDI-II total score revealed a statistically significant difference, with participants not living with HIV presenting higher average scores than those living with HIV (*t* = −2.649, *p* = 0.008, *d* = 0.26). Further details are shown in Table 2.

**Table 2.** Depression Severity (χ² Test) and Continuous BDI-II Scores (t Test) Among gay men Living With and Without HIV.

Measure	Living with HIV (*n* = 217)	Not living with HIV (*n* = 193)	Test statistic	*p*	Effect size
Depression severity					
Minimal	47.9%	37.3%			
Mild	33.2%	35.2%			
Moderate	14.3%	21.2%	χ² = 6.119	0.10	*V* = 0.12
Severe	4.6%	6.2%			
Continuous BDI-II score	11.48 (9.00)	13.92 (9.62)	*t* = −2.649	0.008*	*d* = 0.26

*Note*. Depression severity was tested with χ² (Cramer’s V as effect size). Continuous BDI-II scores were tested with Student’s *t* (Cohen’s d as effect size). Higher scores indicate greater depressive symptoms. *p < 0.05.

To further examine the relationship between serological status and the other main psychological variables, Student’s *t*-tests were conducted to compare homonegativity and perceived stigma between participants living with HIV and those not living with HIV. Table 3 presents the means and standard deviations for each group, as well as the statistical test values, significance levels, and effect sizes.

**Table 3.** Comparison of Homonegativity and Stigma Perception Between gay men Living With and Without HIV.

	Total (*n* = 410)		Living with HIV (*n* = 217)		Not living with HIV (*n* = 193)		*t*	*p*	*d*
Variables	*M*	SD	*M*	SD	*M*	SD	*t*-value	*p*-value	Cohen’s d
Homonegativity (total)	28.21	7.34	25.78	8.83	30.92	3.65	−7.845	0.001*	0.74
Internal perception of stigma	20.35	1.72	17.35	8.28	23.70	3.70	−10.176	0.001*	0.97
External perception of stigma	7.85	1.72	8.41	1.86	7.22	1.28	7.633	0.001*	0.74

*Note*. Higher scores on the homonegativity scale indicate greater internalized negative attitudes toward one’s own sexual orientation; lower scores indicate more affirming attitudes. *p < 0.001.

**Figure 2.**
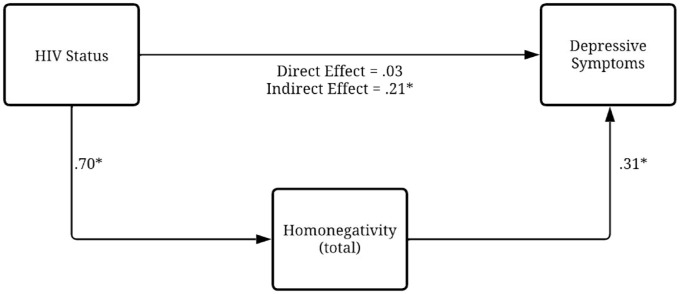
Model of indirect effects of homonegativity in the relationship between HIV and depression. *p < 0.001.

The statistics indicate significant differences in homonegativity and perceived stigma between participants living with HIV and those not living with HIV. For total homonegativity, participants living with HIV scored significantly lower than those not living with HIV (*t* = −7.845, *p* < 0.001, *d* = 0.74, moderate effect size). The same pattern was observed for the internal dimension of homonegativity, with lower scores among participants living with HIV (*t* = −10.176, *p* < 0.001, *d* = 0.97, large effect size). In contrast, for external perception of stigma, participants living with HIV reported significantly higher average scores (*t* = 7.633, *p* < 0.001, *d* = 0.74, moderate effect size; Figure 2).

Subsequently, to test the study’s last hypothesis, analyses of indirect effects were carried out. Results indicated that total homonegativity was statistically associated with the outcome, albeit indirectly. Findings suggested that HIV status showed a positive association with homonegativity (β = 0.70, *p* < 0.001), meaning that people not living with HIV reported significantly higher homonegativity than people living with HIV. Homonegativity was positively associated with depression (β = 0.31, *p* < 0.001), implying that individuals with higher levels of homonegativity tended to report higher levels of depression.

The direct effect of HIV status on depression, after accounting for internalized homonegativity, was not statistically significant (β = 0.03, *p* = 0.69), indicating that living with HIV per se does not predict higher depressive symptoms once internalized homonegativity is considered. The analysis revealed a statistically significant indirect effect through internalized homonegativity (standardized indirect effect = 0.21; 95% CI [0.14, 0.30]), indicating a small to moderate impact. This means that people without HIV had a greater tendency to experience internalized homonegativity, which in turn increased the severity of depressive symptoms. The total effect of HIV status on depression, which combines both the direct and indirect pathways, was statistically significant (β = 0.26, *p* = 0.009), showing that respondents not living with HIV were generally more likely to present depressive symptoms than participants living with HIV.

## Discussion

This study aimed to investigate the prevalence of depressive symptoms and associated factors in Brazilian gay men, comparing the findings between people living with HIV and not living with HIV. Particularly, the investigation explored the links between depression and homonegativity and stigma perception (i.e. internal and external). The results show important findings about the relationship between serological status, depressive symptoms and homonegativity. Among the findings, we highlight that people not living with HIV had higher levels of depressive symptoms compared to those living with HIV, with internal homonegativity emerging as an important variable in understanding this relationship.

### Relationship between depressive symptoms and HIV serological status

The differences observed in depressive symptoms between people living with HIV and people not living with HIV might appear contradictory given previous research that suggested that those with a positive diagnosis would show greater depressive symptoms. Although this finding challenges data from previous investigations (Akena et al., 2010; Javanbakht et al., 2019), it is important to contextualize the current study with the reality of the Brazilian health support system for people living with HIV.

In Brazil’s Unified Health System (SUS), people living with HIV receive structured care, which has been associated with positive mental health outcomes; professional training and guideline resources are examples. One such resource is the *Clinical protocol and therapeutic guidelines for the management of HIV infection in adults*, in which the Ministry of Health emphasizes the importance of starting psychotherapeutic treatment urgently, just as antiretroviral treatment, promulgating the approach of comprehensive care to the patients (Ministério, 2024). It is noteworthy that specialized mental health care can lessen depressive symptoms (Ridout et al., 2024).

In contrast, recent Brazilian studies indicate that people living with HIV may experience higher depressive symptoms when social support is lacking or when stigma is pervasive. In a cross-sectional study with 138 men who have sex with men living with HIV, nearly half of participants (48.5%) presented depressive symptoms, which were significantly correlated with both HIV-related stigma and sexual stigma. Higher depression scores were also associated with lower adherence to antiretroviral therapy (ART; Alckmin-Carvalho et al., 2024a, 2024b).

A larger national survey with 1719 cisgender gay, bisexual, and other MSM living with HIV found that concerns about public attitudes and personalized HIV stigma were positively associated with negative self-image, which in turn was linked to depressive symptoms and poorer ART adherence (Matos et al., 2024). These findings highlight that, while Brazil’s Unified Health System (SUS) offers integrated mental health care within HIV services, structural and psychosocial barriers still influence mental health outcomes. Access to consistent psychological support and targeted stigma-reduction interventions remains critical for improving both mental health and treatment outcomes among people living with HIV.

Post-traumatic growth (PTG) may also help clarify these findings. The concept of post-traumatic growth suggests that individuals may develop resilience, purpose, and coping strategies after adversity. Among participants, living with HIV may have prompted self-care and identity affirmation, contributing to lower depressive symptoms despite social stigma (Moskowitz et al., 2009; Tedeschi and Calhoun, 1996). Meanwhile, people not living with HIV may be more vulnerable to minority stress, that is, a situation in which individuals face overlapping experiences of discrimination (Meyer, 2003) and without access to structured psychological support.

### The role of homonegativity in depression

Relative to the role of homonegativity, this study’s hypothesis was confirmed, suggesting that homonegativity played a significant role in the connection between serological status and depressive symptoms. Those with higher levels of this construct showed higher susceptibility to depression. The cognitive-affective mechanism of minority stress (Hatzenbuehler, 2009) provides one of the main explanations for this association, where internalized stigma can be suggested to function as a chronic psychological stressor, which may contribute to negative cognitive patterns and emotional distress. Gay men who internalize societal attitudes of shame against homosexuality might develop maladaptive beliefs of their self-worth, which can foster continued self-criticism, shame, and emotional distress (Liu and Ren, 2024). Those people may anticipate rejection by others, which increases vigilance and withdrawal from social contact, both implicated in vulnerability to depression (Cisek and Rogowska, 2023).

Internalized homonegativity is another pathway through which it may aggravate the risk of depression by emotional dysregulation. In a study by Lin et al. (2022), they found that gay men with greater internalized homonegativity struggled to control their negative affect when faced with sexual identity stressors. Emotional regulation difficulties were correlated with increased rumination, prolonged sadness, and increased risk for depression episodes. The findings further align with Sommantico and Parrello (2021) who established that emotion regulation mediated the relationship between internalized homonegativity and depression while stressing the need to focus on emotional coping strategies as part of psychotherapeutic interventions.

Homonegativity can undermine coping and increase social avoidance. Although social support is a robust protective factor, individuals with high internalized homonegativity may avoid seeking help, which weakens a key buffer against stress (Jaspal and Breakwell, 2022). Further, Liu and Ren (2024) found that loneliness was the mediating factor between depressive symptoms and internalized homonegativity. These gay men withdraw themselves from potential support systems because of their own high levels of internalized homonegativity, which reinforces persistently negative thought patterns and increases depressive symptoms.

These results underscore the potential relevance of effective mental health interventions that target internalized homonegativity as a primary risk factor in depression. According to Israel et al. (2021), negative beliefs about one’s sexuality might considerably improve symptoms of depression in gay men when approached via cognitive-behavioral approaches. This tells us that self-stigmatizing beliefs can actually be worked on within a therapeutic framework. Additionally, Jaspal and Breakwell (2022) pointed out that identity resilience, which is the ability to hold a positive self view in spite of stigma, is an important predictor of depression or lack thereof. Later findings by Lin et al. (2022) show that therapies need to address both homonegativity reduction and emotional dysregulation support. They found that emotional coping strategies moderated the relationship between internalized homonegativity and depression, such that those who used emotional coping were less likely to experience depression. This indicates that the efficacy of the treatment can be improved through the incorporation of emotion regulation in therapeutic settings.

Finally, we believe that our results demonstrate how operational strengths may interact in the same way as syndemic psychosocial problems, but in the opposite direction. The established relationships with formal health care providers may transform participants’ social consciousness and support, and subsequently, this might be associated with positive mental health and physical health as a result of identity pride, self-esteem, and resilience (Perrin et al., 2020). Consistent with the theories of minority strength, and post-traumatic growth, operational strengths may be an important set of variables minimizing depression symptoms and homonegativity that is still missing from the current HIV behavioral literature, which include factors such as social capital, pre-exposure prophylaxis (PrEP) policies and measures, structural social support, and gay-affirmative approaches (Hart et al., 2018).

### Limitations and future research

Although authors believe that the objectives were met, the limitations of the study were also considered. First, the study assumed a cross-sectional design, which makes it impossible to establish causal relationships between HIV status, depression symptoms and homonegativity. Additionally, the research included a non-probabilistic sample composed of gay Brazilian men with a high level of education (80% of participants had completed higher education or above) and a significantly higher income than the national average. Specifically, 60% of the participants reported earning between four and seven times the minimum wage or more, whereas the majority of the Brazilian population earns considerably less. This socio-demographic profile differs substantially from the broader Brazilian reality, where low income and limited educational attainment are more common. Therefore, generalizations should be made with caution.

In addition, a possible selection bias needs to be addressed. The research was publicized on the social networks of LGBTQIA+ communities, so both digitally excluded individuals and those who do not publicly disclose their sexual orientation and HIV status may be underrepresented. Based on clinical experience and previous research, authors believe that the prevalence of signs and symptoms of depression and homonegativity may be even higher in these cases.

In this study, HIV status was self-reported, which may have had the implication of false negatives or ignorance of serology - although the percentage of individuals who reported living with HIV (15%) in the broader selection of participants, will be quite close to that found in national epidemiological studies. Finally, self-report instruments were used to assess the variables of interest. It is possible to believe that homonegativity, especially internalized homonegativity, may be more difficult to perceive because defense mechanisms, such as denial, may serve to protect individuals from the cognitive dissonance associated with prejudice against themselves. Similarly, symptoms of depression were also assessed by self-report and may have been underestimated by participants’ ability to perceive themselves. Accordingly, for the proper analysis of our results, all these limitations should be taken into account.

Another limitation concerns the recruitment strategies. Participants living with HIV were primarily recruited via snowball sampling within social and community networks, while those not living with HIV were mainly reached through broader social media posts and researchers’ networks. These different strategies may have contributed to systematic sociodemographic differences between the groups (e.g. education, employment, and income), in addition to reflecting structural inequalities faced by people living with HIV in Brazil. Furthermore, the use of online recruitment introduces the possibility of self-selection bias, as individuals who are less digitally connected or less open about their sexual orientation or HIV status may have been under-represented in the sample.

For future directions, suggestions include conducting replication studies with larger and more representative samples of gay men living with and without HIV in Brazil, as well as longitudinal research, which will be crucial to deepen our understanding of the relationship between HIV, homonegativity, and mental health in this population. In addition, it sounds reasonable to suggest that variables that may be associated with better mental health indicators among gay men living with HIV in Brazil, such as the use of health services, including psychotherapy and psychotropic medication are accounted for. Likewise, qualitative studies that investigate, from the perspective of gay men living with HIV, the variables that determine more favorable mental health indicators compared to gay men without HIV could provide unique insights. Future investigations should also employ regression modeling, both in the overall sample and stratified by HIV serostatus, to identify sociodemographic, psychosocial, and stigma-related predictors of depression and homonegativity among Brazilian gay men. Finally, studies that evaluate the factors explored in this investigation among bisexual and transsexual men are of paramount importance in order to cover the specificities of this population.

## Conclusion

More than 50% of the Brazilian gay men who took part in this study reported symptoms of depression at a clinical level, regardless of HIV serologic status. High levels of perceived community homonegativity and internalized homonegativity were also observed. Both symptoms of depression and indicators of homonegativity were lower among gay men living with HIV. Indirect protective mental health mechanisms linked to living with HIV in contemporary Brazil may involve greater access to mental health services, which may foster a stronger sense of community, the formation of self-care strategies and adaptive coping skills to handle challenging situations stemming from social stigma. Notwithstanding lower levels of stigma and depression observed in Brazilian gay men living with HIV, scores in both groups still indicated high, concerning levels. Hence, data suggest that it is crucial to implement public policies to combat stigma, increase access to mental health support, and create targeted interventions for this population.
